# Loss of hepatic autophagy induces α‐cell proliferation through impaired glutamine‐dependent gluconeogenesis

**DOI:** 10.14814/phy2.70381

**Published:** 2025-05-26

**Authors:** Jesse N. Velasco‐Silva, Joseph L. Wilkerson, Daniela Ramos, Hayden K. Low, Faith Bowman, Kimberley J. Evason, Sihem Boudina, William L. Holland, Gregory S. Ducker

**Affiliations:** ^1^ Department of Biochemistry University of Utah Salt Lake City Utah USA; ^2^ Department of Nutrition and Integrative Physiology University of Utah Salt Lake City Utah USA; ^3^ Department of Pathology University of Utah Salt Lake City Utah USA

**Keywords:** alpha cell, autophagy, glucose, hyperglucagonemia, liver metabolism

## Abstract

Autophagy, the highly conserved process of protein and organelle degradation, is suppressed in the liver by obesity and metabolic dysfunction‐associated fatty liver disease and associated with the development of insulin resistance. We generated adult liver‐inducible ATG3 knockout mice (*Atg3* ^*iLKO*^) to characterize pathways linking hepatic autophagy with metabolic homeostasis. Genetic loss of hepatic autophagy leads to a reduction in 16‐h fasted glucose levels, a decrease in endogenous glucose production rates, and an increase in serum amino acids across the fed and fasted states. These changes collectively reflect a loss of hepatic gluconeogenic enzyme activity and not a general inability to degrade amino acids in the liver. Increased circulating glutamine levels resulting from this are associated with an induction of α‐cell hyperplasia, leading to constitutively elevated glucagon levels. However, the loss of hepatic gluconeogenesis renders these animals highly glucagon resistant. Collectively, our data demonstrate that loss of hepatic autophagy is sufficient to activate the hepatic α‐islet cell axis, leading to hyperglucagonemia with impaired glucose production.

## 1 INTRODUCTION

Autophagy is an evolutionarily conserved cellular degradation program vital for maintaining cellular homeostasis. Initiated by the ULK1 complex, autophagosomes form around damaged organelles, proteins, and lipid droplets before fusing with lysosomes for degradation and eventual recycling (Mizushima & Komatsu, 2011). This highly coordinated process requires numerous specialized proteins encoded by autophagy‐related (*Atg*) genes (Cai et al., 2018; Fujita et al., 2008; Komatsu et al., 2005; Mizushima et al., 1998). A critical step in autophagosome expansion involves the lipidation of LC3, a process catalyzed by the ATG3–ATG7 complex (Taherbhoy et al., 2011). Loss of ATG3 is sufficient to inhibit autophagic flux (Cai et al., 2018). Disruption in core autophagy genes is lethal to neonates, and induced global knockout in adult animals leads to neurodegeneration, inflammation, aging, and death (Hara et al., 2006; Inoue et al., 2012; Karsli‐Uzunbas et al., 2014; Kuma et al., 2004).

Impaired autophagic flux is associated with a broad range of cardiometabolic diseases, including heart disease, fatty liver disease, metabolic syndrome, and diabetes (Chen & Lin, 2022; Lavandero et al., 2015; Lin et al., 2019; Yang & Klionsky, 2020). Obesity suppresses essential autophagic proteins like ATG7, disrupting lipophagy, impairing organelle function, inducing lipid droplet accumulation, and causing ER stress, contributing to insulin resistance (Zhang et al., 2018). Restoring autophagy, including lipophagy, improves lipid homeostasis, insulin signaling, and glucose tolerance, emphasizing its crucial role in combating obesity‐induced insulin resistance (Tan et al., 2023; Yang et al., 2010). Acting downstream of the toll‐like receptor 4 (TLR4), autophagy also protects cardiac function by mitigating inflammation, oxidative stress, and cellular dysfunction in obesity‐related cardiac anomalies (Hu & Zhang, 2017). Similarly, defective autophagy in metabolic dysfunction‐associated fatty liver disease contributes to lipid accumulation, organelle damage, inflammation, fibrosis, and progression to severe liver disease (Chen & Lin, 2022; Liu & Czaja, 2013; Zhang et al., 2022).

Studies in model organisms lacking essential autophagic genes have uncovered key roles for liver autophagy in the regulation of metabolic homeostasis. ATG7 is an E1‐like enzyme essential for autophagy, facilitating the conjugation of ATG12 to ATG5, an early step in autophagosome formation (Mizushima et al., 1998). Hepatocyte‐specific inducible *Atg7*‐knockout mice are hypoglycemic upon fasting due to impaired gluconeogenesis (Ezaki et al., 2011; Toledo et al., 2018). In fact, when Atg7 loss is induced globally, mice cannot survive prolonged fasting due to severe hypoglycemia (Karsli‐Uzunbas et al., 2014). Ezaki et al. and Karsli‐Uzunbas et al. proposed that this was due to impaired substrate production, as they observed decreased serum amino acids upon prolonged fasting in ATG7 global and liver‐specific‐KO animals, respectively (Ezaki et al., 2011; Karsli‐Uzunbas et al., 2014). In contrast, Toledo and colleagues observed reduced expression of key gluconeogenic genes (*Pepck*) and identified that this was in response to the loss of the clock cryptochrome repressor‐1 (CRY1) (Toledo et al., 2018). shRNA‐induced loss of ATG7 results in insulin resistance in C57BL/6 mice and reduced glucose clearance, but it was not reported whether fasting glucose levels were also repressed (Yang et al., 2010). It remains unclear whether reduced gluconeogenic flux in hepatic autophagy‐deficient animals also induces global changes in glucose regulation, for example, insulin or glucagon resistance.

Overexpression of *Atg7* in the liver of *ob/ob* mice or those subjected to a high‐fat diet simultaneously improved whole‐body insulin sensitivity and signaling, leading to enhanced glucose uptake by peripheral tissues and reduced hepatic glucose production (Sadeghi et al., 2023; Yang et al., 2010). Together, these studies demonstrate an essential role for hepatic autophagy in the process of gluconeogenesis and, when absent, lead to systemic alterations in glucose and insulin regulation. However, a comprehensive metabolic workup quantifying glucose production and disposal throughout the organism has not been described for a liver autophagy‐deficient animal.

In this study, we sought to identify mechanisms that would connect changes in liver autophagy with the systemic regulation of metabolism by studying hepatocyte‐specific inducible *Atg3* knockout mice. We applied a quantitative approach to investigate changes in systemic insulin and glucagon metabolism that resulted from the loss of autophagic flux. We found that the loss of hepatic autophagy suppresses gluconeogenesis without impacting the catabolism of amino acids or leading to systemic insulin resistance. However, due to impaired liver gluconeogenesis, glutamine levels were highly elevated, leading to the activation of pancreatic alpha cells and hyperglucagonemia.

## 2 RESULTS

### 2.1 Liver‐specific *Atg3* ^*iLKO*^ mice have decreased fasting glucose and increased serum amino acid levels

**FIGURE 1:**
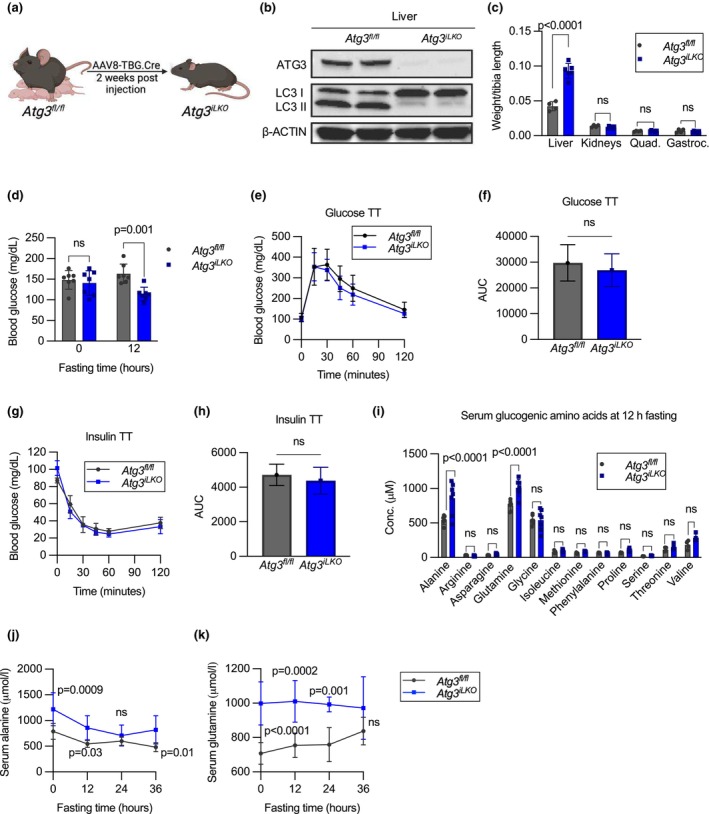
Liver‐specific *Atg3* ^*iLKO*^ mice have decreased fasting glucose and increased amino acid levels. (a) Overview of the liver‐specific autophagy‐deficient mouse model for this study (*Atg3* ^*iLKO*^). (b) Western blots of ATG3, LC3I, and II proteins in liver tissue, with β‐actin as loading control. (c) Tissue weight normalized to tibia length of 16 h fasted mice (mean ± SD, *n* = 4–6, *p* < 0.0001 by two‐way ANOVA). (d) Blood glucose levels after 12 h fasting (mean ± SD, *n* = 7, *p* < 0.001 by two‐way ANOVA). (e) Glucose tolerance test and (f) area under the curve during a 120‐min challenge (mean ± SD, *n* = 7–10, *p* < 0.05 by unpaired *T*‐test). (g) Insulin tolerance test and (h) area under the curve for 120‐min challenge (mean ± SD, *n* = 5–7, *p* < 0.05 by unpaired *T*‐test). (i) Serum glucogenic amino acids after a 12 h fast in *Atg3* ^*fl/fl*^ and *Atg3* ^*iLKO*^ mice (mean ± SD, *n* = 6–7, *p* < 0.0001 by two‐way ANOVA). Serum (j) alanine and (k) glutamine during 36 h of fasting (mean ± SD, *n* = 6–7, *p* < 0.0001 by two‐way ANOVA).

To investigate the interplay between liver autophagy and systemic glucose metabolism in adult mice, we generated an inducible liver‐specific autophagy‐deficient mouse (*Atg3* ^*iLKO*^) by administering AAV8‐TBG‐Cre virus to 8‐week‐old *Atg3* ^*fl/fl*^ mice (Figure 1a) (Cai et al., 2018). *Atg3* ^*fl/fl*^ animals were injected with AAV‐TBG‐null virus to generate sibling controls. Two weeks post‐injection, we confirmed liver‐specific loss of ATG3 protein expression and observed a reduction in the levels of cleaved light‐chain 3‐II (LC3‐II) by Western blot after a 16 h fast, indicating a loss of autophagosomes (Figure 1b and Figure S1a). Two weeks after the induction of Cre, whole‐body composition as measured by NMR was unaltered (Figure S1b). *Atg3* ^*iLKO*^ animals displayed hepatomegaly with protein accumulation and mild microvesicular steatosis consistent with reports from *Atg7* knockout mice (Figure 1c and Figure S1c–e) (Karsli‐Uzunbas et al., 2014). When challenged with a 12 h fast, *Atg3* ^*iLKO*^ animals had reduced serum glucose (Figure 1d). However, upon extended fasting (36 h), both control and *Atg3* ^*iLKO*^ maintained body weight equally, and serum glucose levels were no longer different (Figure S1f,g).

To understand whether decreased glucose levels during fasting were due to changes in glucose disposal, we performed a glucose tolerance test (GTT) on 12 h fasted mice and observed no difference in whole‐body glucose clearance (Figure 1e,f). *Atg3* ^*iLKO*^ animals had increased levels of fasted insulin (pre‐GTT) but similar increases in insulin as control animals upon glucose challenge (Figure S1h). To assess whether the changes in fasting insulin were indicative of altered insulin sensitivity, we performed an insulin tolerance test (ITT) and observed no differences in blood glucose levels between groups (Figure 1g,h). Together, these results show that reduced fasting glucose levels in *Atg3* ^*iLKO*^ mice were not due to peripheral insulin sensitivity and/or enhanced glucose disposal.

We next investigated whether glucose production was impaired in fasted *Atg3* ^*iLKO*^ animals. To test if liver glycogen released was reduced, we stained liver sections from 16 h fasted mice with Periodic acid–Schiff (PAS) stain. PAS staining was reduced in fasted liver from both control and knockout mice, although to a lesser extent in *Atg3* ^*iLKO*^ hepatocytes, which showed clear hypertrophy (Figure S1I). Total liver glycogen in fasted *Atg3* ^*iLKO*^ mice as assayed by a fluorometric test was not different compared to controls, suggesting that glycogen breakdown was minimally impacted (Figure S1j). In contrast, serum levels of major gluconeogenic amino acids (alanine and glutamine) were elevated in fasted animals (Figure 1i). Differences in circulating concentrations of other gluconeogenic amino acids were not statistically significant, although most trended higher in *Atg3* ^*iLKO*^ animals. Alanine and glutamine levels were elevated even in the fasted state of *Atg3* ^*iLKO*^ mice and remained significantly above control animals over a 24 h fast with no evidence of sex differences (Figure 1j,k, and Figure S1k,l). Conversely, non‐amino acid gluconeogenic substrates glycerol and lactate were unchanged in *Atg3* ^*iLKO*^ serum (Figure S1m,n). The consistently elevated levels of serum gluconeogenic amino acids alanine and glutamine suggest that *Atg3* ^*iLKO*^ animals have a defect in gluconeogenesis.

### 2.2 Loss of hepatic autophagy impairs endogenous glucose production by suppressing gluconeogenic enzyme expression

**FIGURE 2:**
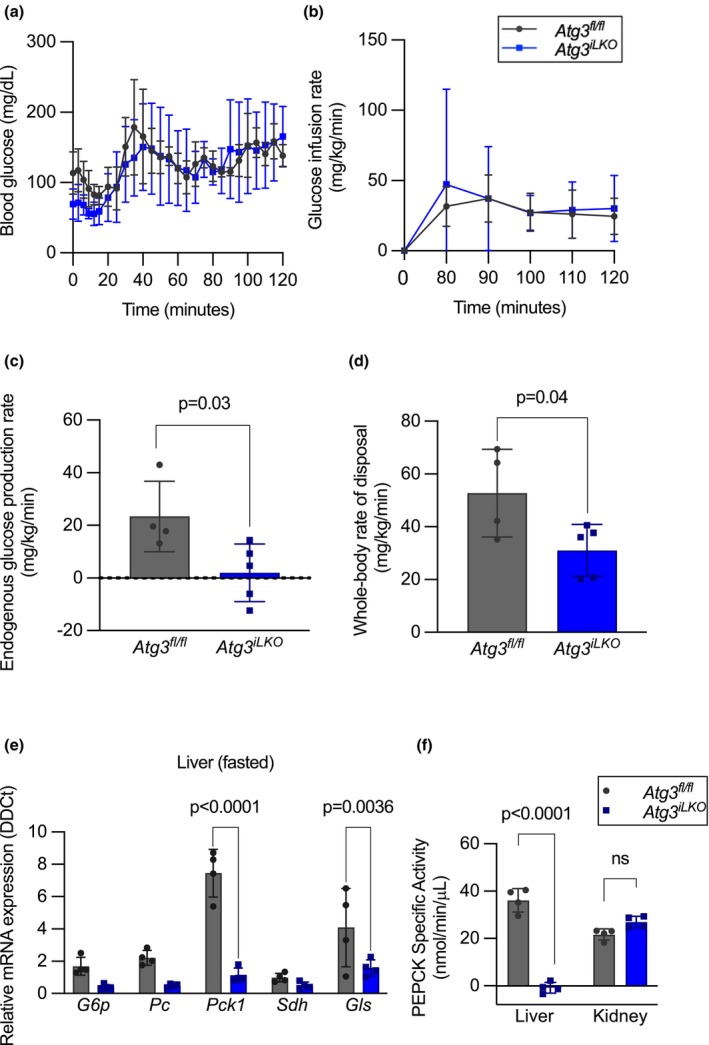
Loss of hepatic autophagy impairs endogenous glucose production by suppressing gluconeogenic enzyme expression. (a) Blood glucose levels, (b) glucose infusion rate, (c) endogenous glucose production rates, and (d) whole‐body rate of glucose disposal during euglycemic clamp (mean ± SD, *n* = 4, *p* < 0.05 by unpaired *T*‐test). (e) Relative mRNA expression of glucose‐6‐phosphatase (*G6p*), pyruvate carboxylase (*Pc*), phosphoenolpyruvate carboxykinase (*Pck1*), succinate dehydrogenase (Sdh), and glutaminase (*Gls*) genes in the livers of *Atg3* ^*fl/fl*^ and *Atg3* ^*iLKO*^ mice upon 16 h fast (mean ± SD, *n* = 4, *p* < 0.0001 by Sidaks multiple comparison test). (f) Phosphoenolpyruvate carboxykinase activity in the liver and kidney of fasted mice (mean ± SD, *n* = 4, *p* < 0.0001 by two‐way ANOVA).

To establish that loss of *Atg3* impairs glucose production in vivo, we performed hyperinsulinemic‐euglycemic clamps in 16 h fasted *Atg3* ^*iLKO*^ and *Atg3* ^*fl/fl*^ control mice. Insulin was administered at 2 μL/min and blood glucose clamped at 120–130 mg/dL by continuous variable glucose administration (Figure 2a). Glucose consumption and production fluxes were quantified by constant co‐infusion of [U‐^13^C]‐glucose and enrichment fractions determined by liquid chromatography‐mass spectrometry (LC–MS). Euglycemia was maintained with a final glucose infusion rate in *Atg3* ^*iLKO*^ mice that was slightly higher (16%) than in controls (Figure 2b, Figure S2a). Using tracer enrichment data, we calculated endogenous glucose production rates for both *Atg3* ^*iLKO*^ and control mice (Figure 2c) (Sharma et al., 2018). Glucose production was suppressed in *Atg3* ^*iLKO*^ animals and glucose disposal rates were significantly reduced (Figure 2d). In summary, loss of liver autophagy severely impaired the endogenous glucose production rate but was in part compensated for by a reduction in peripheral glucose disposal.

To characterize the mechanisms by which *Atg3* ^*iLKO*^ suppressed glucose production, we performed immunoblotting to quantify hepatic insulin signaling. After a 16 h fast, we observed elevated phosphorylation of the insulin receptor (IR) in the liver from *Atg3* ^*iLKO*^ mice treated with the hyperinsulinemic‐euglycemic clamps (Figures S2b,c and S5b). This suggests that *Atg3* ^*iLKO*^ livers are insulin‐sensitive and may help to suppress gluconeogenic enzyme expression. We then quantified gluconeogenic gene expression in the liver and kidney by qRT‐PCR in fed and fasted mice. Within the liver, expression of core gluconeogenic genes (glucose‐6‐phosphatase (*G6p*), pyruvate carboxylase (*Pc*), and phosphoenolpyruvate carboxykinase (*Pck1*)) was dramatically reduced in both the fasted and fed states in *Atg3* ^*iLKO*^ livers but unaltered in kidney (Figure 2e and Figure S2d–f). As was reported in *Atg7* KO mice, *Cry1* gene expression was reduced in fed *Atg3* ^*iLKO*^ mice (Figure S2g) (Toledo et al., 2018). To confirm that gluconeogenesis was functionally impaired in the liver, we performed a PEPCK activity assay on liver and kidney lysates from fasted control and *Atg3* ^*iLKO*^ animals. Using a pyruvate substrate, we confirmed that gluconeogenesis was inhibited in *Atg3* ^*iLKO*^ liver lysates but unchanged in kidney (Figure 2f).

### 2.3 Glutamine is uniquely unable to stimulate glucose production in *Atg3* ^*iLKO*^ mice

**FIGURE 3:**
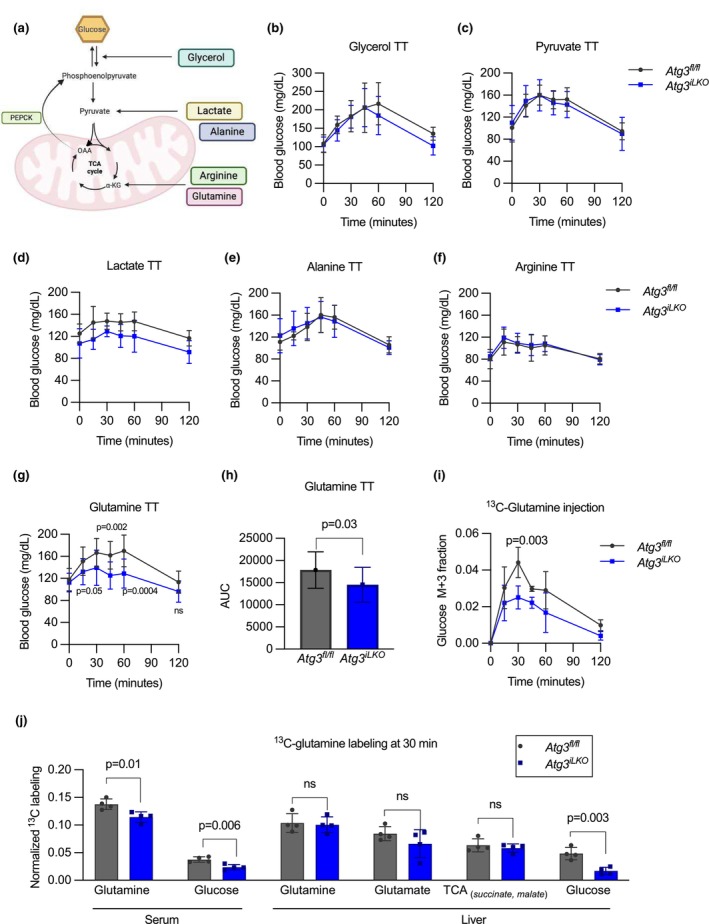
Glutamine is uniquely unable to stimulate glucose production in *Atg3* ^*iLKO*^ mice. (a) Multiple pathways for metabolites to support gluconeogenesis. (b) Glycerol tolerance test (*n* = 7 for *Atg3* ^*fl/fl*^ and *n* = 11 for L‐ *Atg3* ^*iLKO*^). (c) Pyruvate tolerance test (*n* = 14 for *Atg3* ^*fl/fl*^ and *n* = 16 for *Atg3* ^*iLKO*^). (d) Lactate tolerance test (*n* = 5 for *Atg3* ^*fl/fl*^ and *n* = 6 for *Atg3* ^*iLKO*^). (e) Alanine tolerance test (*n* = 7 for *Atg3* ^*fl/fl*^ and *n* = 9 for *Atg3* ^*iLKO*^). (f) Arginine tolerance test (*n* = 5 for *Atg3* ^*fl/fl*^ and *n* = 7 for *Atg3* ^*iLKO*^). (g) Glutamine tolerance test. (h) Glutamine area under the curve (mean ± SD, *n* = 12, *p* < 0.0001 by Šídák's multiple comparisons test). (i) Labeling fraction of serum during a 25% [U‐^13^C]‐glutamine tolerance test (*n* = 3 for *Atg3* ^*fl/fl*^ and *n* = 4 for *Atg3* ^*iLKO*^). (j) Fractional labeling of liver metabolites 30 minutes after [U‐^13^C]‐glutamine tolerance test (mean ± SD, *n* = 3–4, *p* < 0.0001 by two‐way ANOVA). All the mice used for these tolerance tests were fasted for 16 h.

To better understand the nature of the substrate‐selective defect in gluconeogenesis and potential compensation by the kidney, we performed nutrient tolerance tests of major glucogenic substrates (Figure 3a). Glycerol, pyruvate, lactate, alanine, and arginine all increased circulating blood glucose levels by similar amounts in *Atg3* ^*iLKO*^ and control animals (Figure 3b–f and Figure S3a–e). In contrast, when administered glutamine, *Atg3* ^*iLKO*^ mice were unable to increase circulating glucose levels to the same degree as controls (Figure 3g,h). We repeated the glutamine tolerance test with [U‐^13^C]‐glutamine to track the fate of glutamine carbon atoms. In *Atg3* ^*fl/fl*^ control animals, [U‐^13^C]‐glutamine bolus led to the production of 3‐labeled glucose (M + 3) with the same kinetics as the unlabeled tolerance test, demonstrating the direct gluconeogenic effects of glutamine carbon. This increase was attenuated in *Atg3* ^*iLKO*^ mice (Figure 3i).

We next examined livers from mice 30 min after injection with [U‐^13^C]‐glutamine. Total serum ^13^C tracer enrichment was decreased in *Atg3* ^*iLKO*^ mice, implying elevated metabolite turnover. Liver [U‐^13^C]‐glutamine labeling was not different in mice lacking autophagy, suggesting enhanced glutamine uptake in this organ. The metabolism of glutamine into liver glutamate and TCA cycle products succinate and malate was also not different between genotypes (Figure 3j). Consistent with our tracing data, liver glutaminase (*Gls*) expression was not different in the fasted state and actually increased in the fed state (Figure S2d). However, liver glucose labeling from glutamine was decreased by 3‐fold, highlighting a defect in gluconeogenesis. Tracing data showed no differences in glutamine metabolism in muscle (quadricep and gastrocnemius) and kidney in *Atg3* ^*iLKO*^ mice (Figure S4a–c). Overall, our single‐dose glutamine tracing data suggest a specific functional defect in hepatic gluconeogenesis that is independent of TCA cycle activity.

### 2.4 Hepatic TCA cycle flux is maintained in *Atg3* ^*iLKO*^ mice

**FIGURE 4:**
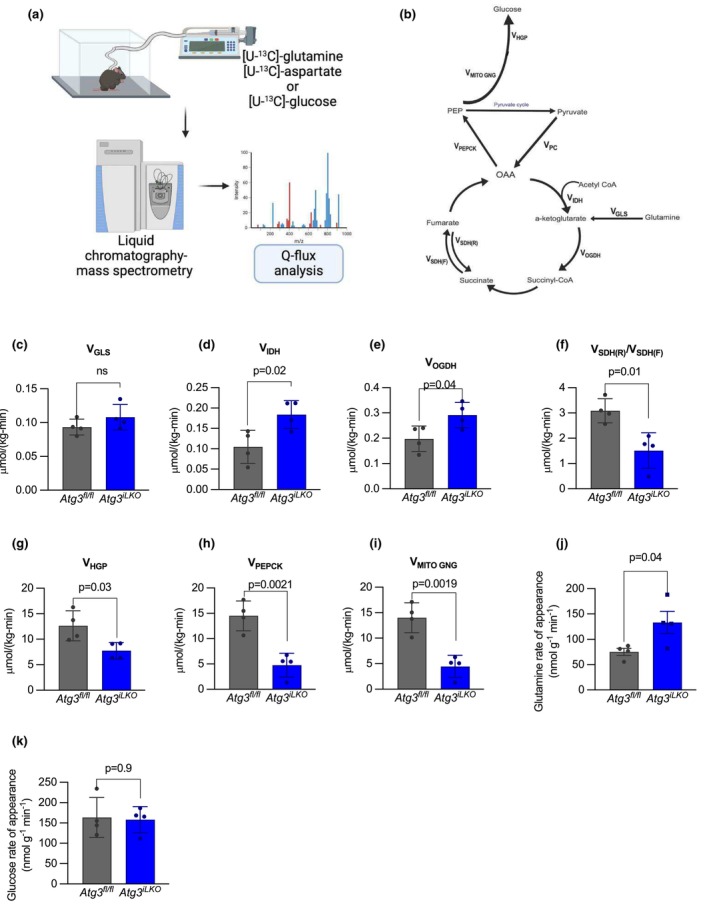
Glutamine tracing reveals that hepatic gluconeogenesis but not glutaminase fluxes are suppressed in *Atg3* ^*iLKO*^ mice. (a) Schematic for the quantification of TCA cycle fluxes by isotope tracer infusion, LC–MS measurement, and integrated Q‐flux analysis. (b) Relative and absolute rates of gluconeogenic and TCA enzyme fluxes were obtained using the Q‐flux method. (c) Glutaminase (V_GLS_) absolute fluxes in fasted *Atg3* ^*fl/fl*^ and *Atg3* ^*iLKO*^ mice. (d) Isocitrate dehydrogenase (V_IDH_), (e) α‐Ketoglutarate dehydrogenase (V_OGDH_), and (f) net succinate dehydrogenase (V_SDH(R)_/V_SDH(F)_) relative rates. (g) Hepatic glucose production, (h) phosphoenolpyruvate carboxykinase (V_PEPCK_), and (i) mitochondrial gluconeogenesis (V_MITOGNG_) absolute rates (*n* = 4 for each group). (j) [U‐^13^C]‐glutamine and (k) [U‐^13^C]‐glucose rate of appearance in 16‐h fasted *Atg3* ^*fl/fl*^ and *Atg3* ^*iLKO*^ mice. Data are means ± SD, *n = 4, p* < 0.05 by unpaired *T*‐test).

To understand the totality of metabolic re‐wiring in autophagy deficient animals, and if specific enzymatic steps were impaired, we performed additional steady‐state metabolic labeling experiments to quantify hepatic TCA fluxes. We performed stable isotope infusions in fasted mice with jugular vein catheters and assessed liver TCA metabolite enrichment. Isotope enrichment data were then analyzed using the published Q‐flux method to determine TCA cycle fluxes (Hubbard et al., 2023). This approach provides a comprehensive quantification of in vivo mouse hepatic metabolism by algebraic integration of isotopic enrichment data from steady‐state infusions of [U‐^13^C]‐glutamine and [U‐^13^C]‐aspartate combined with [U‐^13^C]‐glucose determined rates of hepatic glucose production to calculate rates of TCA cycle anaplerotic metabolism and TCA turning (Figure 4a,b).

Consistent with our gene expression data, glutaminase flux (V_GLS_) was unchanged, indicating that there was no specific defect in hepatic glutamine catabolism (Figure 4c). Oxidative TCA metabolism, represented by rates of isocitrate dehydrogenase (V_IDH_) and α‐Ketoglutarate dehydrogenase (V_OGDH_) was increased, and the net succinate dehydrogenase (V_SDH(R)_/V_SDH(F)_) was decreased in *Atg3* ^*iLKO*^ animals compared to *Atg3* ^*fl/fl*^ (Figure 4d–f). We estimated total hepatic glucose production (V_HGP_) from systemic [U‐^13^C]‐glucose infusion and observed a 39% reduction in *Atg3* ^*iLKO*^ mice (Figure 4g). This may represent an overestimate as the method assumes a negligible contribution by the kidneys, which may not be true in these animals. Using glutamine labeling data and this flux value, we determined that hepatic gluconeogenic fluxes were significantly reduced in *Atg3* ^*iLKO*^ animals (V_PEPCK_ and V_MITOGNG_) consistent with our observations from clamp experiments (Figure 4h,i). Our in vivo flux data provide strong evidence that the *Atg3* knockout liver remains competent to metabolize glutamine into the TCA cycle to produce gluconeogenic precursors, and that, in fact, TCA cycle turning is increased, but that diminished gluconeogenesis is at the root of defective glucose homeostasis.

Interestingly, we observed that the serum glutamine rate of appearance was significantly higher in *Atg3* ^*iLKO*^ mice compared to control mice (Figure 4j). This was not true of glucose (Figure 4k). As fasting glutamine levels were increased in *Atg3* ^*iLKO*^ mice, we can deduce that rates of consumption were also increased, indicating enhanced glutamine turnover flux in *Atg3* ^*iLKO*^ mice. As glutamine was unable to generate glucose in these animals effectively, we propose that this reflects enhanced glutamine to glutamate futile cycling.

### 2.5 *Atg3* ^*iLKO*^ mice have α*‐*islet cell hyperplasia and hyperglucagonemia

**FIGURE 5:**
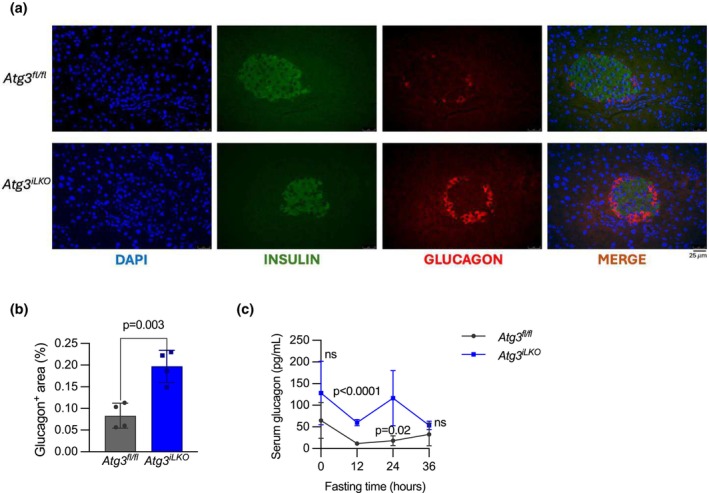
α‐Islet cell hyperplasia leads to hyperglucagonemia. (a) Representative immunohistochemical staining images of fasted pancreas from *Atg3* ^*fl/fl*^ and *Atg3* ^*iLKO*^ mice. (b) Glucagon‐positive area in the pancreas of fasted *Atg3* ^*fl/fl*^ and *Atg3* ^*iLKO*^ mice (mean ± SD, *n* = 4, *p* < 0.05 by unpaired *T*‐test). (c) Serum glucagon concentration during a 36 h fast (mean ± SD, *n* = 4, *p* < 0.0001 by Šídák's multiple comparisons test)

Several groups have demonstrated that increased glutamine levels are sufficient to drive an increase in α‐cell number, leading to enhanced glucagon secretion (Dean et al., 2017; Holst et al., 2017; Solloway et al., 2015). Therefore, we investigated whether increased levels of serum glutamine due to the loss of liver autophagy also augmented pancreatic α‐cell number. We performed immunohistochemical staining of glucagon in the islets of the pancreas isolated from fasted *Atg3* ^*iLKO*^ and control mice (Figure 5a). Pancreatic islets from *Atg3* ^*iLKO*^ mice showed a 2.4‐fold higher glucagon‐positive area compared to control mice (Figure 5b). We then measured total glucagon in the serum of fasted mice and found that it was elevated over the course of a 24‐h fasting period compared to control mice (Figure 5c). To reconcile hyperglucagonemia with our observed defects in glucose production, we again examined liver signaling in *Atg3* ^*iLKO*^ mice.

### 2.6 *Atg3* ^*iLKO*^ mice are glucagon‐resistant

**FIGURE 6:**
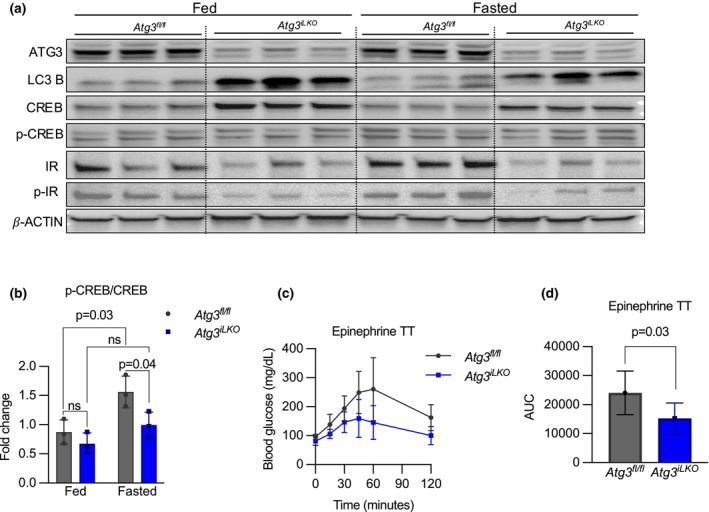
*Atg3* ^*iLKO*^ mice are glucagon‐resistant. (a) Western blot analysis and (b) quantification of p‐CREB/CREB protein in liver tissue of fed and fasted *Atg3* ^*fl/fl*^ and *Atg3* ^*iLKO*^ mice (mean ± SD, *n* = 3, *p* < 0.0001 by two‐way ANOVA). (c) Epinephrine tolerance test (mean ± SD, *n* = 3, *p* < 0.0001 by two‐way ANOVA) and (d) area under the curve (mean ± SD, *n* = 4–6, *p* < 0.05 by unpaired *T*‐test).

To examine glucagon signaling in *Atg3* ^*iLKO*^ animals, we performed immunoblotting to quantify glucagon‐induced phosphorylation of the PKA substrate CREB (Zhao et al., 2023). Total levels of CREB were elevated in both fed and fasted *Atg3* ^*iLKO*^ animals compared to controls, but p‐CREB levels failed to increase in *Atg3* ^*iLKO*^ animals during fasting (Figure 6a,b, Figure S5a), suggesting primary glucagon resistance. To examine if this resistance manifested systematically, we performed an epinephrine tolerance test to measure glucagon‐stimulated glucose production (Stumvoll et al., 1999). As shown in Figure 6c,d, a bolus of epinephrine was unable to stimulate the production of glucose in *Atg3* ^*iLKO*^ animals. In summary, absent hepatic autophagy, liver cells are glucagon resistant, and animals show a systemic inability to increase glucose production upon stimulus.

## 3 DISCUSSION

In this study, we show that loss of hepatic autophagy profoundly disrupts liver gluconeogenesis, resulting in systemic metabolic dysregulation. Two weeks after *Atg3* loss, mice showed impaired systemic glucose homeostasis during short‐term fasting and increased hepatic insulin sensitivity, leading to reduced overnight glucose levels, decreased endogenous glucose production, and elevated serum alanine and glutamine. Consequently, *Atg3* knockout in the liver disrupts glutamine‐dependent gluconeogenesis, resulting in elevated circulating glutamine levels, α‐cell hyperplasia, and constitutive hyperglucagonemia.

Our findings further support hypoglycemia as a hallmark of defective hepatic autophagy. Previous studies have shown hypoglycemia in liver‐specific *Atg7* knockout mice, and we now report the same in liver‐specific *Atg3* knockout animals (Ezaki et al., 2011; Toledo et al., 2018; Yang et al., 2010). The precise molecular mechanisms behind the suppression of gluconeogenesis in autophagy‐deficient mice remain unclear. Toledo et al. identified CRY1 protein, a circadian clock regulator, as a master repressor of gluconeogenic genes, and when no longer degraded in *Atg7* knockout mice, accumulates, preventing gluconeogenesis. As the protein negatively regulates its own gene expression, *Cry1* mRNA expression is suppressed in autophagy‐deficient animals (Figure S2f). We additionally observed changes in liver signaling pathways that contribute to the suppression of gluconeogenesis. We observed enhanced activation of the insulin receptor when given insulin and constitutive suppression of glucagon‐dependent PKA signaling, as shown by suppressed phospho‐CREB. Together, these suggest a rewiring of the hepatic signaling network to further suppress gluconeogenic enzyme expression.

In animals with both whole‐body and liver‐specific deletion of *Atg7* and *Atg5*, circulating serum arginine levels were significantly reduced (Poillet‐Perez et al., 2018). In contrast, in our *Atg3* ^*iLKO*^ mice, fasted arginine was unchanged and arginine tolerance remained normal. In the prior work, the decrease in arginine levels was attributed to the release of hepatic Arg1, indicating liver damage. Our lack of an arginine phenotype suggests that the loss of *Atg3* and the time point we chose for our study result in less liver damage than prior studies. Instead, we observed a marked increase in serum glutamine levels in both fed and fasted *Atg3* ^*iLKO*^ mice, highlighting a selective alteration in amino acid handling that was downstream of inhibition of gluconeogenesis. This large increase in glutamine was associated with α‐islet cell hyperplasia and hyperglucagonemia.

The interplay between serum amino acids and pancreatic α‐cells is vital for glucose homeostasis, with amino acids serving as both key metabolic substrates and critical signaling molecules that regulate hormonal responses crucial for energy balance during nutrient deprivation. This interaction forms a feedback loop sensing nutrient availability and modulating glucagon secretion, thereby maintaining metabolic stability (Holst et al., 2017). However, livers lacking autophagy are no longer responsive to glucagon, and this feedback loop is broken. Increases in serum glutamine by other mechanisms can also lead to enhanced glucagon levels and stimulate gluconeogenesis. Mice with reduced hepatic glutaminase 2 (GLS2), a key enzyme in glutamine metabolism, have elevated fasting plasma glucagon and glutamine levels, coupled with lower fasting blood glucose in insulin‐resistant conditions. In critically ill patients, glutamine supplementation alone has been shown to significantly reduce the need for exogenous insulin to maintain glycemic control. Similarly, in individuals with type 2 diabetes, glutamine supplementation has been reported to lower postprandial blood glucose levels, improving overall glucose homeostasis (Grau et al., 2011; Laviano et al., 2014).

We were surprised to observe that hepatic autophagy impaired gluconeogenesis only from glutamine. In mice, the reported major gluconeogenic substrates are lactate, glutamine, and glycerol, and earlier studies describe a central role in liver gluconeogenesis for all of these (Stumvoll et al., 1999). Our enzyme expression data and reaction data from lysates firmly rule out any possible residual gluconeogenic flux from our *Atg3* ^*iLKO*^ livers, despite the ability of the liver TCA cycle to incorporate glutamine carbon. While our study focuses on liver‐specific autophagy loss, the kidney is an underappreciated second major site of gluconeogenesis (Stumvoll et al., 1998). We believe our findings highlight the kidney's ability to at least partially compensate for impaired hepatic gluconeogenesis, as evidenced by the maintenance of some glucose production in *Atg3* ^*iLKO*^ mice. Additionally, although our systemic glutamine tracing data did not reveal a clear block in glutamine utilization by the kidney, the limited compensation may be due to slow glutamine uptake or low glutaminase enzyme activity in renal tissue. Further studies examining kidney‐specific metabolic adaptations, enzyme expression, and substrate handling in this context would help clarify its role in maintaining glucose homeostasis during hepatic autophagy deficiency.

Our study highlights a mechanism by which hepatic autophagy regulates glucose metabolism through the glucagon to α‐cell signaling axis and suggests a bidirectional regulation between liver autophagy and systemic insulin and glucagon signaling. Future studies will seek to uncover substrate‐specific sites of gluconeogenesis in mouse tissues and the role of autophagy loss downstream of metabolic dysfunction in signaling to the pancreatic islet. Our current study is limited by the potential of ATG3‐specific, autophagy‐independent effects, its restriction to adult hepatocytes, and the absence of systemic impacts observed in whole‐body knockout models. Our observations in young healthy mice may not recapitulate the effects of hepatic autophagy loss on obese or aged individuals.

## 4 MATERIALS AND METHODS

### 4.1 Animals and animal care

All procedures were approved by the Institutional Animal Care and Use Committee (IACUC) of the University of Utah. Mice were housed at 22–23°C using a 12 h light/12 h dark cycle. Animals were maintained on a standard chow diet with ad libitum access (ENVIGO, Teklad Diets. 2020X). Food access was only restricted during experiments (fasted state). Mice had ad libitum access to water at all times. Generation of adult liver‐specific ATG3‐knockout mice was performed by retroorbital injection of 3.5e11 GC of pAAV.TBG.PI.Null.bGH (Addgene# 105536‐AAV8) or AAV.TBG.PI.Cre.rBG (Addgene# 107787‐AAV8) to 6–8 week old *Atg3* ^*fl/fl*^ mice. The age of mice used for all studies was 8–13 weeks old (2–4 weeks post‐AAV8 administration). Equal numbers of male and female littermates were used in the studies. No animals were excluded from any experiments.

### 4.2 Analysis of body composition

Lean and fat mass were determined via TD‐NMR (LF50, Bruker) in live 8–10 week‐old mice.

### 4.3 Jugular vein catheterization surgery (JVC)

A jugular vein catheter (JVC) was implanted in mice under anesthesia using best‐practice aseptic technique. Sterile mouse catheters were purchased from SAI Infusion Technologies (MJC‐01), and one channel vascular access button from Instech Laboratories, Inc. (VABM1B/25). A dissection microscope (Leica S9i Stereo) was utilized during the placement of the catheter. Mice were allowed a 5 to 7‐day period to recover normal activity and behavior after the surgical procedure before further experimentation.

### 4.4 Hyperinsulinemic‐euglycemic clamp

Hyperinsulinemic‐euglycemic clamps were performed on conscious pre‐catheterized and unrestrained 16 h fasted mice, as previously described (Holland et al., 2011; Kusminski et al., 2012). In brief, a constant infusion of 200 mM [U‐^13^C]‐glucose (Cambridge Isotope Laboratories Inc.; CLM‐1396) for 120 min prior to the clamp and throughout the duration of the experiment allowed for calculations of glucose kinetics. Hyperinsulinemia was initiated with a primed infusion of insulin 25 μL per 75 s (Lilly; NDC 0002–8215‐17), then a continuous insulin infusion of 2 μL/min, while a variable infusion of 50% dextrose (Fisher Scientific; D16) for 120 min allowed for the achievement of a targeted blood glucose between 120 and 130 mg/dL.

### 4.5 Western blot analysis

Protein concentration from tissue homogenate was determined by using the Pierce BCA protein assay kit (Thermo Scientific; 23,227) per the manufacturer's instructions. Equal concentrations of denatured protein were resolved by SDS‐PAGE and transferred to nitrocellulose membranes (BioRad; 1,704,158). Rabbit primary antibodies used in this study were against ATG3 (Cell Signaling; 3415S), LC3B (Cell Signaling; 2775S), CREB (Cell Signaling; 4820), p‐CREB (Invitrogen; PA1‐851), GAPDH (Cell Signaling; 2118), IR (Cell Signaling; 4B8), p‐IR (Cell Signaling; 3024), AKT (Cell Signaling; 9272), p‐AKT‐S473 (Cell Signaling; D9E), and p‐AKT‐T308 (Cell Signaling; D25E6). Equal loading was confirmed using rabbit monoclonal anti‐beta actin antibody (Cell Signaling; 4970S). Goat anti‐rabbit poly‐horseradish peroxidase (Invitrogen; 32,260) was used as the secondary antibody. Peroxidase activity was detected using the SuperSignal Western Pico PLUS chemiluminescent substrate (Thermo Scientific; 34,580).

### 4.6 Tolerance tests

Tolerance tests were performed in mice 2–3 weeks post AAV8 injection after a 16 h fast. Basal glucose was determined via a tail nick prior to intraperitoneal (i.p.) administration of glucose 25 g/kg body weight (Fisher Scientific; D16), glycerol 1 g/kg body weight (Sigma; G5516), pyruvate 1 g/kg body weight (Sigma; P5280), glutamine 1 g/kg body weight (Sigma; G8540), alanine 1 g/kg body weight (Sigma; A7469), arginine 1 g/kg body weight (Sigma; A5131), lactate 1 g/kg body weight (Sigma; 1,614,308), insulin 0.75 IU/kg body weight (Lilly; NDC 0002–8215‐17), or epinephrine 3.75 μL/g body weight (PAR pharmaceutical; NDC 42023–168‐01). Subsequent blood glucose values were measured after 0, 15, 30, 60, and 120 min with a glucometer (Contour Next EZ, Contour Next). [U‐^13^C]‐glutamine (Cambridge Isotope Laboratories Inc.; CLM‐1822) lethal tolerance test: 16 h fasted mice were intraperitoneally (IP) injected with 1 mg/kg of a mixture of [U‐^13^C]‐glutamine (25%) and ^12^C glutamine (75%) diluted in sterile saline. Thirty minutes following the injection, blood was collected by tail snip (~10 μL) and transferred into blood collection tubes (Microvette CB 300 Z), and mice were sacrificed by cervical dislocation to collect and snap freeze tissue.

### 4.7 In vivo infusions

JVC mice were housed on a standard light cycle (6 am–6 pm). At the beginning of the experiment, mice were transferred to new cages without food around 6 pm (the beginning of their awake cycle) and infused the next day (16 h fasting). The infusion setup (Instech Laboratories) included a swivel and tether to allow the mouse to move around the cage freely. Water‐soluble [U‐^13^C]‐glucose (200 mM), [U‐^13^C]‐glutamine (100 mM), or [U‐^13^C]‐aspartate (100 mM) (Cambridge Isotope Laboratories; CLM‐1396, CLM‐1822, and CNLM‐544) was prepared as solutions in sterile normal saline. The infusion rate was set to 0.1 μL/ min^−1^*g^−1^ for 120–180 min. Blood was collected by tail snip (~10 μL) and transferred into blood collection tubes (Microvette CB 300 Z). Blood samples were stored on ice and then centrifuged at 5000 × *g* for 5 min at 4°C to collect serum. Tissue harvest was performed at the end of the infusion after euthanasia by cervical dislocation. Tissues were quickly dissected, rinsed in cold PBS, clamped with a precooled Wollenberger clamp, and dropped in liquid nitrogen.

### 4.8 LC–MS analysis of polar metabolites

Extracted polar metabolite samples were analyzed by LC‐MC. Separation was achieved by hydrophilic interaction liquid chromatography (HILIC) using a Vanquish HPLC system (ThermoFisher Scientific). The column was an Xbridge BEH amide column (2.1 mm × 150 mm, 2.5 μM particular size, 130 Å pore size, Waters Co.) run with a gradient of solvent A (20 mM ammonium hydroxide, 20 mM ammonium acetate in 95:5 water: acetonitrile, pH 9.45) and solvent B (100% acetonitrile) at a constant flow rate of 150 uL/min. The gradient function was: 0 min, 90% B; 2 min, 90% B; 3 min, 75% B; 7 min, 75% B; 8 min, 70% B; 9 min, 70% B; 10 min, 50% B; 12 min, 50% B; 13 min, 25% B; 14 min, 25% B; 16 min, 0% B; 20.5 min, 0% B; 21 min, 90% B; 25 min, 90% B. Autosampler temperature was 4°C, column temperature 30°C, and injection volume 3 μL. Samples were injected by electrospray ionization into a QExactive QE Plus orbitrap mass spectrometer (Thermo Fisher Scientific) operating in negative ion mode with a resolving power of 70,000 at m/z of 200 and a full scan range of 75–1000 m/z. Data were analyzed using the EL‐MAVEN software package and specific peaks assigned based on exact mass and comparison with known standards (Melamud et al., 2010). Extracted peak intensities were corrected for natural isotopic abundance (Su et al., 2017).

### 4.9 Metabolite extraction of serum

Serum (4 μL) was added to 140 μL of −80°C 80% methanol/20% water, and vortexed with 72 μL of chloroform on ice. Following phase separation, the aqueous extract was centrifuged at 15,060 × *g* for 10 min at 4°C, and the supernatant was transferred to clean tubes for LC–MS analysis.

### 4.10 Metabolite extraction of tissue

30 mg of frozen tissue was pulverized by a Cryomill (Retsch; 20.749.0001) using liquid nitrogen as coolant. Ground tissue was then mixed with 525 μL of −80°C 40:40:20 methanol:acetonitrile:water with 0.1% formic acid (extraction solvent). Samples were briefly vortexed before neutralizing with 8 μL of 15% ammonium bicarbonate per 100 μL of extraction solvent. The extract was then vortexed and centrifuged at 15,060 × *g* for 10 min at 4°C; the supernatant was collected, and the pellet was mixed with 525 μL of −80°C 40:40:20 methanol:acetonitrile:water. The second extraction was then vortexed and centrifuged at 15,060 × *g* for 10 min at 4°C; supernatants were combined, and 525 μL of chloroform was added. The mix was vortexed and centrifuged at 15,060 × *g* for 10 min at 4°C; the final supernatant was transferred to LC–MS tubes for analysis.

### 4.11 Insulin and glucagon levels

Serum insulin levels were measured using the mouse insulin ELISA kit (Crystal Chem; 90,080) using mouse standards according to the manufacturer's guidelines. Glucagon levels in serum were measured by the glucagon ELISA assay kit (Mercodia; 10–1281‐01) using mouse standards according to the manufacturer's guidelines.

### 4.12 Glycogen levels

Glycogen levels in mice livers were measured using the Glycogen Assay Kit (Sigma‐Aldrich; MAK016) according to the manufacturer's guidelines.

### 4.13 Phosphoenolpyruvate carboxykinase activity

Phosphoenolpyruvate carboxykinase activity was measured using a colorimetric assay kit (Sigma‐Aldrich; MAK408) following the manufacturer's guidelines.

### 4.14 Histology and immunohistochemistry

For histology, pancreas tissues were fixed in 10% buffered formalin, embedded in paraffin, sectioned at 5 μm, and stained with Periodic acid–Schiff (PAS). Immunohistochemistry was performed on paraffin‐embedded pancreas sections stained with insulin polyclonal antibody (Thermo Fisher Scientific # PA1‐26938), glucagon polyclonal antibody (Thermo Fisher Scientific # PA5‐88091), and DAPI. After overnight incubation, slides were washed with PBS and incubated for 1 h with Alexa Flour 488 Guinea pig (Jackson ImmunoResearch Inc.; 706,545,148) and Alexa Flour 647 Anti‐Rabbit (Invitrogen; A31573). Imaging was performed using a Leica DM6000B fluorescence microscope at 40× magnification. Glucagon‐positive areas from the average of 10 islets per mouse pancreas were quantified with ImageJ software.

### 4.15 RNA extraction

RNA extraction was performed using the miRNeasy mini kit (Qiagen; 217,004) as per the manufacturer's instructions. Snap‐frozen tissue (20–30 mg) was homogenized using the CryoMill homogenizer (Retsch; 20.749.0001). The RNA was eluted in 30 μL RNase‐free water. RNA concentration was confirmed by using the NanoDrop One (Thermo Fisher Scientific).

### 4.16 Quantitative reverse transcription PCR (RT‐qPCR)

For RT‐qPCR, RNA was extracted as described above. cDNA was generated from 1 μg of RNA using the qScript™ cDNA SuperMix (QuantaBio; 95,048) on a T100 thermal cycler (BioRad) according to the manufacturer's instructions. Omission of Reverse Transcriptase and a template‐free reaction were used as negative controls. Quantitative real‐time PCR was performed with the PowerTrack™ SYBR Green Master Mix for qPCR (Thermo Fisher Scientific; A46109) and primers from Integrated DNA Technologies (Table S1) targeting glucose‐6‐phosphatase (*G6p*), pyruvate carboxylase (*Pc*), phosphoenolpyruvate carboxykinase (*Pck1*), succinate dehydrogenase (Sdh), glutaminase (*Gls*), and cryptochrome 1 (*Cry1*) using a LightCycler 480 (Roche) in a 96‐well plate setting (final reaction volume 10 μL per well). Each biological replicate (mouse) was run in duplicate, and β‐actin was used as a housekeeping gene for normalization.

### 4.17 Statistical analysis

Data were plotted as the mean ± SD. Student's *t*‐test or two‐way. ANOVA was carried out using Prism (GraphPad Prism), and statistical significance was considered meaningful at *p* < 0.05.

## AUTHOR CONTRIBUTIONS

J.N.V‐S. and G.S.D. were involved in conceptualization and investigation. J.N.V‐S., J.L.W., D.R., H.L., F.B., K.E., W.L.H., and G.S.D. were involved in methodology. J.N.V‐S., W.L.H., and G.S.D. were involved in analysis. J.N.V‐S., S.B., W.L.H., and G.S.D. were involved in writing and editing. G.S.D was involved in funding acquisition.

## CONFLICT OF INTEREST STATEMENT

The authors declare no conflicts of interest.

## ETHICS STATEMENT

This study was conducted in accordance with ethical guidelines governing general laboratory practice including transperancy, full disclosure and integrity. The use of animal models was approvied by and followed guidelines established by the Institutional Animal Care and Use Committee of the University of Utah.

## Supporting information


Figures S1–S5.

Table S1.

## Data Availability

Requests for information, resources, and reagents can be sent to the lead contact, Gregory Ducker (greg.ducker@biochem.utah.edu). Unique materials and reagents generated as a result of this study are available from the lead contact upon request.
