# Supplementary material for: Loss of hepatic autophagy induces α‐cell proliferation through impaired glutamine‐dependent gluconeogenesis
Source: Physiol Rep. 2025 May 26;13(10):e70381. doi: 10.14814/phy2.70381 (PMC12106947; doi:10.14814/phy2.70381)
Supplement: Supplementary file 1 — Figures S1–S5. [file PHY2-13-e70381-s001.zip › PHYSREP-2025-01-043-f01-z-.pdf]

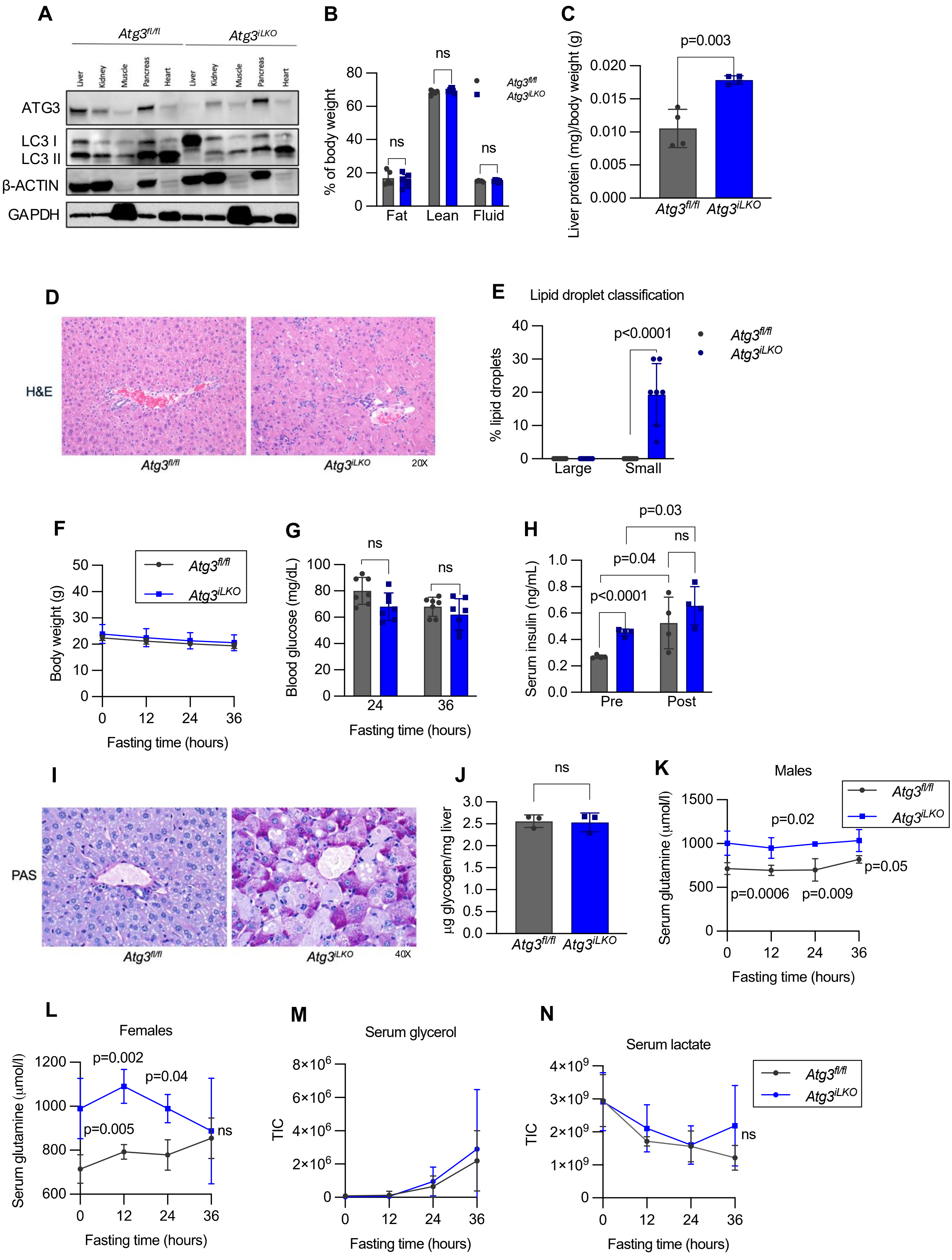


Supplemental Figure 1. *Atg3*$^{iLKO}$ mouse phenotyping.

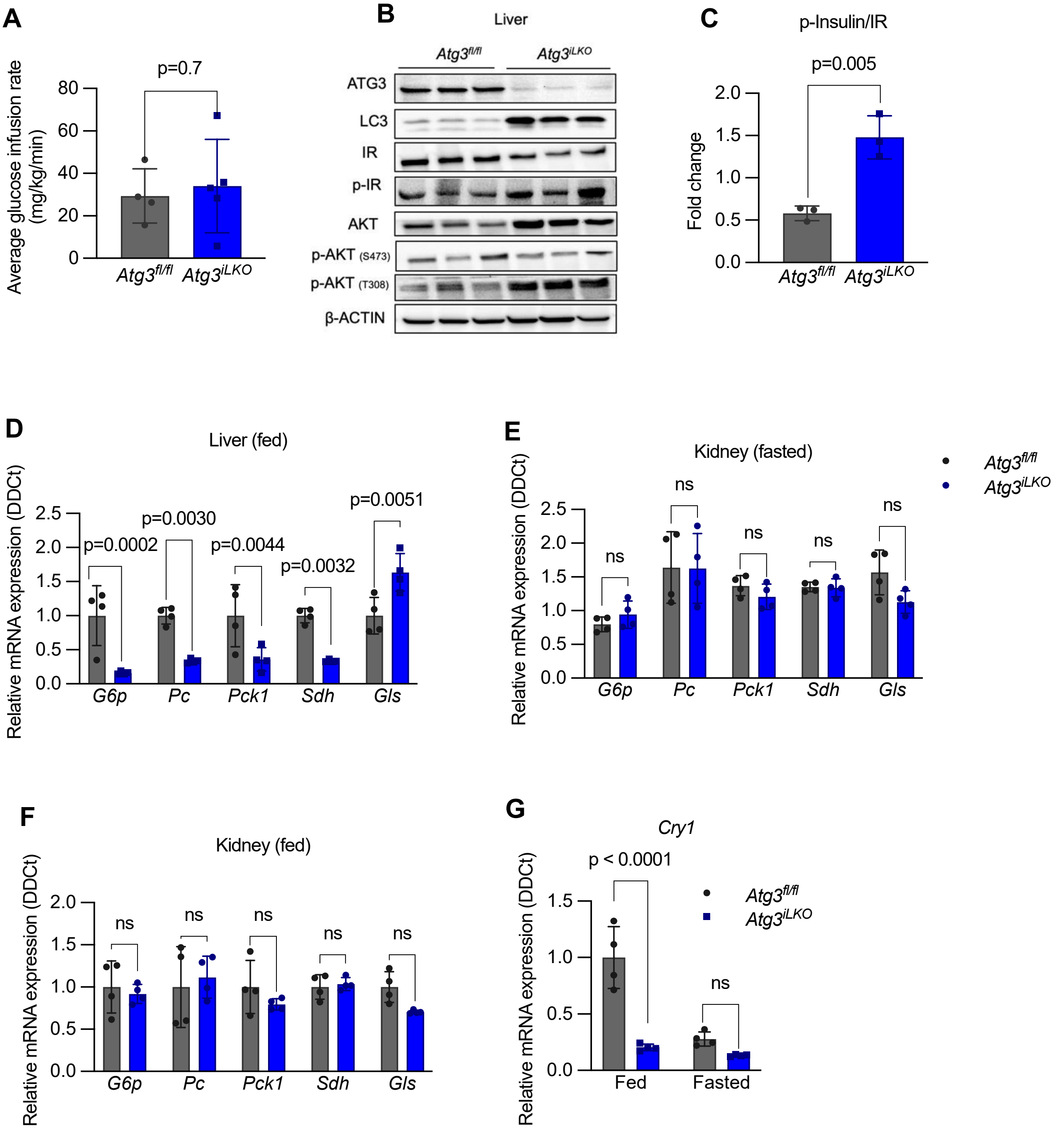


Supplemental Figure 2. *Atg3*$^{iLKO}$ mice have liver insulin receptor activation .

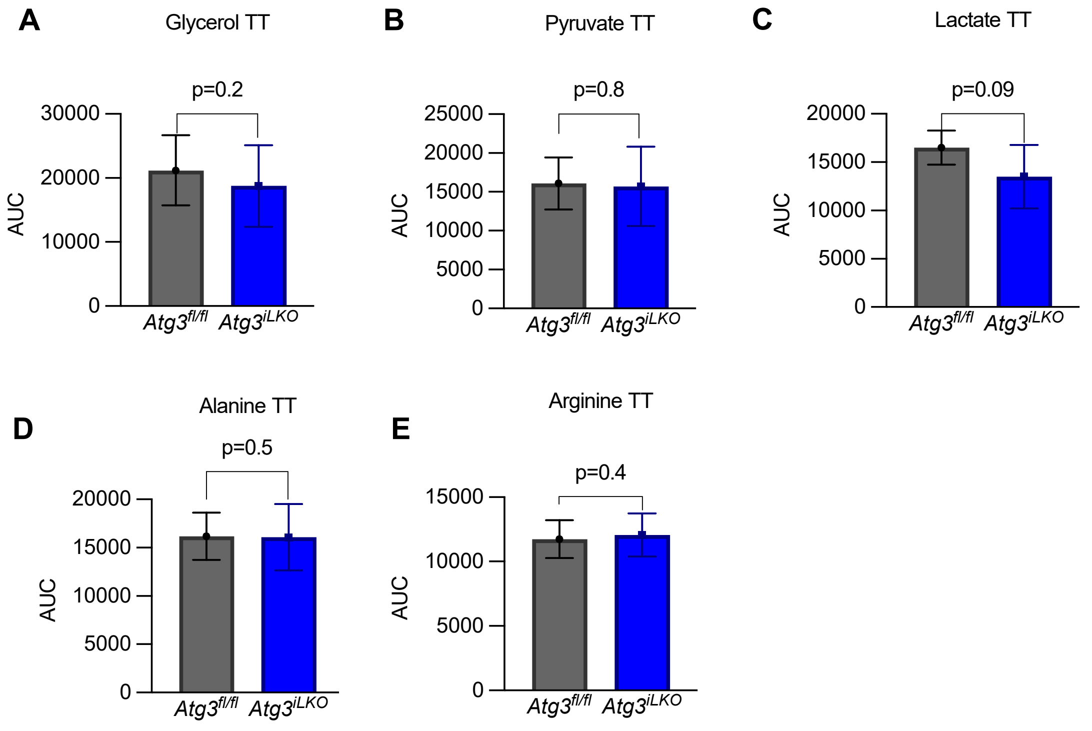


Supplemental Figure 3. Nutrient tolerance test area under curve.

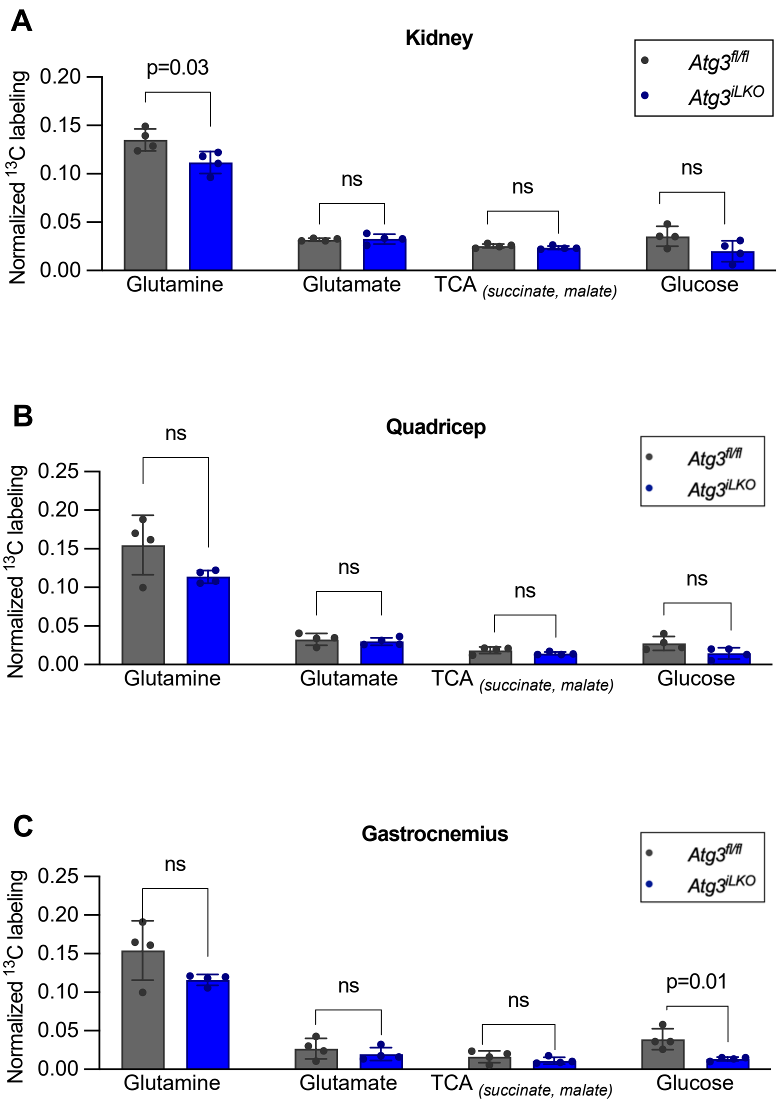


Supplemental Figure 4. [U-$^{13}$C]-glutamine tolerance test.

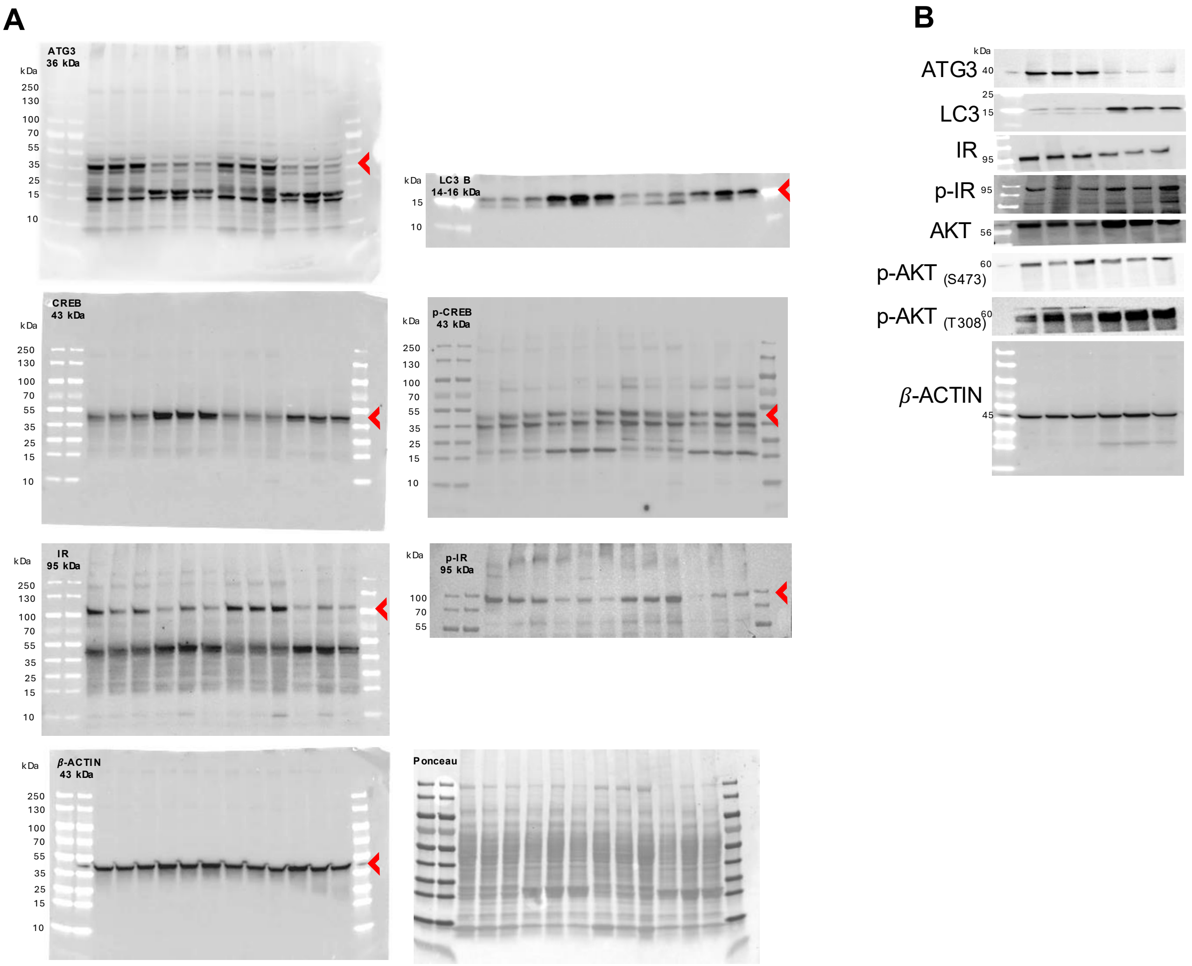


Supplemental Figure 5. Complete western blot images.
