# Supplementary material for: Loss of hepatic autophagy induces α‐cell proliferation through impaired glutamine‐dependent gluconeogenesis
Source: Physiol Rep. 2025 May 26;13(10):e70381. doi: 10.14814/phy2.70381 (PMC12106947; doi:10.14814/phy2.70381)
Supplement: Supplementary file 1 — Figures S1–S5. [file PHY2-13-e70381-s001.zip › Supplemental_Figures_captions.docx]

**Supplemental Figure** **1. *Atg3^iLKO^* mouse phenotyping**. **(A)** Western blot of *Atg3^fl/fl^* and *Atg3^iLKO^* mouse liver, kidney, muscle, and heart showing liver-specific Atg3 knockout. **(B)** Whole-body fat, lean, and fluid composition by TD-NMR (mean ± SD*, n* *=* *5–7,* *p* < 0.0001 by two-way ANOVA). **(C)** Total liver protein (mg) normalized to body weight (g) in Atg3f/f and *Atg3^iLKO^* (mean ± SD, *n* *=* *4,* *p* < 0.05 by unpaired T-test). **(D)** Hematoxylin and eosin (H&E) staining of livers from 16 h fasted *Atg3^fl/fl^* and *Atg3^iLKO^* mice. **(E)** Liver lipid droplet quantification in *Atg3^fl/fl^* and *Atg3^iLKO^* animals. **(F)** *Atg3^fl/fl^* and *Atg3^iLKO^* body weight during a 36 h fast. **(G)** Blood glucose levels during 24 and 36 h fasts (for **E** and **G** means ± SD*, n* *=* *7,* *p* < 0.0001 by two-way ANOVA). **(H)** Serum insulin pre- and post-glucose tolerance test (mean ± SD*, n* *=* *4,* p < 0.0001 by two-way ANOVA). **(I)** Periodic acid-Schiff staining of fed and fasted livers from *Atg3^fl/fl^* and *Atg3^iLKO^* mice. **(J)** Total glycogen (μg) normalized to liver mass (mg) in fasted mice (mean ± SD, *n* *=* *3,* *p* < 0.05 by unpaired T-test). Serum glutamine concentration in **(K)** male and **(L)** female mice during a 36 h fast. **(M)** Total ion count (TIC) of serum glycerol and **(N)** lactate in *Atg3^fl/fl^* and *Atg3^iLKO^* mouse during a 36 h fast (mean ± SD*, n* *=* *7,* *p* < 0.0001 by two-way ANOVA).
**Supplemental Figure 2. *Atg3^iLKO^* mice have liver insulin receptor activation.** **(A)** Average glucose infusion rate during a 120-minute clamp (*n* = 4 for *Atg3^fl/fl^* and *n* = 5 for *Atg3^iLKO^*). **(B)** Western blot analysis of the insulin signaling pathway and **(C)** p-insulin/IR fold change in the liver tissue of mice subject to hyperinsulinemic-euglycemic clamp (for **A** and **C** mean ± SD, *n* *=* *3,* *p* < 0.05 by unpaired T-test). **(D-F)** Relative mRNA expressions of glucose-6-phosphatase (*G6p*), pyruvate carboxylase (Pc), phosphoenolpyruvate carboxykinase (*Pck1*), succinate dehydrogenase (*Sdh*), and glutaminase (*Gls*) in the **(D)** fed liver, **(E)** fasted and **(F)** fed kidney of *Atg3^fl/fl^* and *Atg3^iLKO^* mice. **(G)** cryptochrome 1 (*Cry1*) gene in the fed and fasted liver of *Atg3^fl/fl^* and *Atg3^iLKO^* mice (for **D-G** means ± SD*, n* *=* *4,* *p* < 0.0001 by two-way ANOVA).
**Supplemental Figure 3. Nutrient tolerance test area under the curve**.**(A)** Glycerol (*n* = 7 for *Atg3^fl/fl^* and *n* = 11 for L- *Atg3^iLKO^*), **(B)** pyruvate (*n* = 14 for *Atg3^fl/fl^* and *n* = 16 for *Atg3^iLKO^*), **(C)** lactate (*n* = 5 for *Atg3^fl/fl^* and *n* = 6 for *Atg3^iLKO^*), **(D)** alanine (*n* = 7 for *Atg3^fl/fl^* and *n* = 9 for *Atg3^iLKO^*), **(E)** arginine (n = 5 for *Atg3^fl/fl^* and n = 7 for *Atg3^iLKO^*) tolerance test area under the curve. Data are means ± SD*,* *p* < 0.05 by unpaired T-test.
**Supplemental Figure 4. [U-^13^C]-glutamine tolerance test**. Normalized ^13^C labeling in **(A)** kidney, **(B)** quadricep, and **(C)** gastrocnemius muscle metabolites after 30 minutes of [U-^13^C]-glutamine tolerance test. Data are means ± SD*, n* *=* *4,* *p* < 0.0001 by two-way ANOVA.
**Supplemental Figure 5. Complete western blot images.** Related to Figure 6A. **(B)** Related to Supplemental Figure 2B.
