# Supplementary material for: Loss of hepatic autophagy induces α‐cell proliferation through impaired glutamine‐dependent gluconeogenesis
Source: Physiol Rep. 2025 May 26;13(10):e70381. doi: 10.14814/phy2.70381 (PMC12106947; doi:10.14814/phy2.70381)
Supplement: Supplementary file 2 — Table S1. [file PHY2-13-e70381-s002.docx]

|  | |  |  |  |  |  |
| --- | --- | --- | --- | --- | --- | --- |
| **Gene** | **Forward primer** | | | **Reverse primer** |  |  |
| *Pck1* | TATGCTGATCCTGGGCATAAC | | | TCATGGCCAAGTTAGTCTTCC |  |  |
| *Gls* | GGTGGTTTCTGCCCAATTAC | | | CTGCCCTGAGAAGTCATACA |  |  |
| *Pc* | ACCTACGGCTTCCCTATTATCT | | | CGGGTGTAATTCTCTTCCAACT |  |  |
| *Sdh* | GCTGGAGAAGAATCGGTTATGA | | | GCATCGACTTCTGCATGTTTAG |  |  |
| *Cry1* | GAAATATGGCGTTCCTTCCCT | | | CGTGTAAGTGCCTCAGTTTCT |  |  |
| *G6p* | CGTATGGATTCCGGTGTTTGA | | | GAAAGTGAGCAGCAAGGTAGA |  |  |
| *B-actin* | GACAGGATGCAGAAGGAGATTAC | | | TCAGTAACAGTCCGCCTAGAA |  |  |

Supplemental Table 1. qPCR primer sequences
